# A bibliometric and visual analysis of research trends and hotspots of myocardial apoptosis: A review

**DOI:** 10.1097/MD.0000000000035236

**Published:** 2023-09-22

**Authors:** Kun Lian, Xin Li, Xiaoyi Wang, Fei Wang, Meng Yang, Jiahao Ye, Lin Li, Zhixi Hu

**Affiliations:** a Hunan University of Chinese Medicine, Changsha, China.

**Keywords:** bibliometric analysis, cardiomyocyte apoptosis, CiteSpace, traditional Chinese medicine, visual analysis

## Abstract

**Background:**

Recent studies have found that cardiomyocyte apoptosis is closely associated with the pathophysiological development of various cardiovascular diseases, for example chronic heart failure and myocardial infarction. At present, there are many researches in this field, such as pharmacological research, traditional Chinese medicine intervention research and pathway research. However, the relevant research is fragmented, with few comprehensive analysis and systematic combing.

**Methods:**

The relevant literature on cardiomyocyte apoptosis was downloaded from the Web of Science Core Collection (WoSCC) and PubMed databases. Citespace 6.1.R2 software Microsoft Excel 2019 and VOSviewer1.6.18.0 were used for bibliometric and visual analysis of publication volume, countries, institutions, journals, authors, keywords.

**Results:**

Since 1996, there are 1881 research articles and reviews related to cardiomyocyte apoptosis published by 10,313 researchers from 1648 institutions in 58 countries or regions were included. The number of annual publications showed an upward trend, especially in recent years. Countries participating in this research area include China, the United States, and Japan. Capital Medical University, Harbin Medical University are the key research institution, and other institutions also have substantial contribution on the project as to cardiomyocyte apoptosis. The journal EUR REV MED PHARMACO has a large number of publications, whereas CIRCULATION has the highest number of co-citations. Keywords analysis showed that apoptosis, expression and oxidative stress had higher frequencies, leading to 8 clusters.

**Conclusions:**

Cardiomyocyte apoptosis is a hot research field in recent years. Through visualization and bibliometric analysis, it is found that this field focus on hotspots like clinical manifestations including heart failure or myocardial infarction, and microscopic mechanisms such as oxidative stress and inflammation.

## 1. Introduction

Apoptosis, which is also referred to programmed cell death, is the spontaneous and orderly death of cells controlled by genes to maintain homeostasis.^[1]^ Recent studies demonstrated that cardiomyocyte apoptosis is closely related to etiology of heart failure and other cardiovascular diseases.^[2–4]^ Numerous studies have shown that increased apoptosis increases the risk of myocardial infarction and heart failure, and that limiting apoptosis protects the heart.^[5]^ One of the major consequences of HF after myocardial infarction is massive cell apoptosis, which represents one of the critical molecular mechanisms by which HF causes death. Morita H suggested that p53, for example, induces the apoptosis of cardiomyocytes in failing hearts.^[6]^ Petrovic et al^[7]^ proposed that apoptosis of cardiomyocytes can cause sporadic loss of cardiomyocytes and, and in cases of extensive occurrence, might cause heart failure.

At present, emerging global research organizations are mainly focused on exploring pathways and targets research,^[8,9]^ investigating the potential of traditional Chinese medicine intervention research,^[10,11]^ and conducting pharmacological research.^[12–14]^ Despite the large amount of experimental and clinical data and literatures have been published over the past 27 years, the relevant studies are fragmented, with few systematic reviews and comprehensive analyses. Therefore, collecting and analyzing data from publications is helpful for researchers to grasp the current research progress and understand the research status and development frontiers in this research area.

Bibliometrics, previously termed as Statistical Bibliography, was first introduced in 1969. The research proves that CiteSpace could fully reveal the objective situation of scientific development.^[15]^ Bibliometrics in conjunction with visualization processing of CiteSpace and VOSviewer more intuitively presents the research frontiers and development trends of related fields.

Our research intents to comprehensively recognize the research status of cardiomyocyte apoptosis by analyzing the literature published in the past 27 years. And it also designed to analyze, interpret and summarize the relevant literatures to illustrate future research hotspots and directions, and provide certain reference and guidance for researchers in the future scientific research and clinical practice.

## 2. Methods

### 2.1. Search strategies

On August 22, 2022, literatures on myocyte apoptosis were searched in Science Network Core Collection and Pubmed. All retrieved articles were exported as “full records and cited literatures” and downloaded to a text file named download*txt. Cardiomyocyte apoptosis, myocardial apoptosis or myocardium cell apoptosis were retrieved as the main topic words respectively. The retrieval method is advanced retrieval, and the retrieval time starts from the database establishment. The language of the article is English. The first and second authors read the title, abstract, and full text of all articles and then delete articles that are not relevant to the topic. If they disagree, consult the third author.

### 2.2. Data inclusion and exclusion

1) Inclusion criteria: Studies related to cardiomyocyte apoptosis were classified as articles and review.

2) Exclusion criteria: repeated publication of literature, other types of literature, literature with incomplete data information, literature that is not recognized by the software.

3) Processing process: The first and second authors read the title, abstract, and full text of all articles and then delete articles that are not relevant to the topic. If they disagree, consult the third author.

### 2.3. Data collection and analysis

Import the above text files to the built-in data converter of CiteSpace 6.1.R2 software to generate data formats that can be recognized by the software and save them to corresponding folders. Create a project in CiteSpace called apoptosis, and set the following parameters: Time slicing = January 1996 to December 2022, years per slice = 1, Selection criteria = Top 50, Pruning: Pathfinder, Pruning Tender Networks, the rest keep the default Settings. Node type areas according to the research purpose, select “country, institution, author, keyword, cited author.” Select the keyword Burst Analysis from the submenu.

In general, by each node on the map represents an author, word, or institution, With node size indicating the frequency of occurrence or mention. The colored nodes correspond to specific years, while the colored circles extending outward from the nodes represent the time range from 1996 to 2022. In addition, the lines connecting nodes depict cooperative, co-existing, or co-entrant relationships.^[16,17]^ Centrality is used to measure the importance of nodes within a network. The node with higher centrality indicate more frequent connections with other nodes, thereby suggesting their greater importance in the whole network.^[18,19]^

According to the annual publication data generated by CiteSpace, copy and save the data into Microsoft Office Excel 2019 to make the data analysis chart. VOSviewer1.6.18.0 software parameter: more than 5 articles published in the journal. The total citations of the journal exceed 20 times. The number of publications by the author is more than 4 papers. The author has been cited no less than 20 times. The keyword appears at least 20 times. Retain the default values for other parameters.

## 3. Results

### 3.1. General information and annual publication output

**Figure 1.**
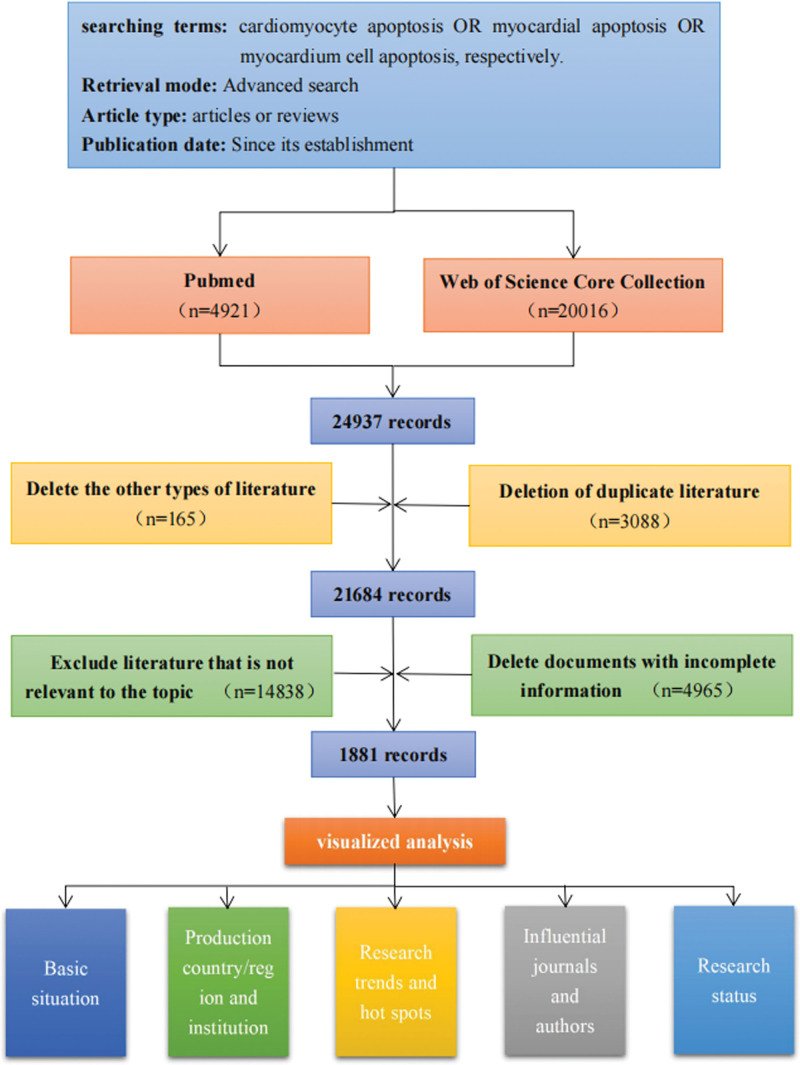
Flowchart of literature selection. Nodes represent corresponding research objects, and the larger the number of publications, the larger the radius of nodes. The lines between nodes represent the cooperative relationship between the research objects, and the closer the cooperative relationship is, the thicker the lines are. The color from cool to warm indicates the age from distant to recent.

We retrieved 24,937 publications including 4921 articles in PubMed and 20,016 articles in Web of Science Core Collection. After deleting duplicated and irrelevant literature, 1881 literature were finally included. The selection flow chart of literatures is detailed in Figure 1.

**Figure 2.**
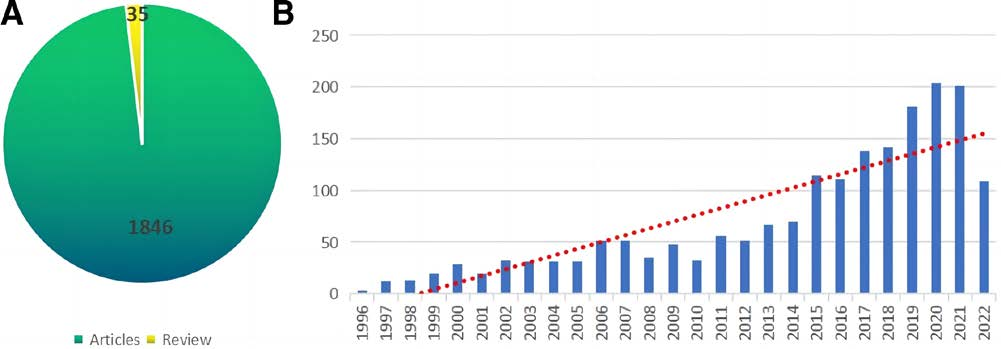
(A) Literature types of myocyte apoptosis. (B) Annual number of publications on myocyte apoptosis from 1996 to 2022.

The progress of subject knowledge is reflected by the number of publications each year, which also serves as an important indicator of trends.^[20,21]^ In the histogram of Figure 2B, the number of annual publications in the field was summarized over the past 27 years. From 1996 to 2022, the number of publications showed an overall growth trend.

The changing trend of published literature in this field might be roughly categorized into 2 phases. The first phase, stretching from 1996 to 2014, showed gradual and steady growth. The second phase, from 2015 to 2022, shows a significant growth trend. Among them, there were more than 100 in 2015 for the first time, and more than 200 in 2020. Articles published in 2022 is also expected to reach more than 200. From the general trend, this field is developing rapidly and receiving more and more attention, especially in the past 8 years, which has entered a period of rapid development. In addition, articles accounted for more than 98% of the literature types (Fig. 2A), indicating that this research field pays more attention to original research.

### 3.2. Distribution of countries/territories and institutions

The collaboration chart can show the cooperation relationship between countries or institutions in the field of this research area, thus offering new guidance for assessing the academic impact of the country or institution. It helps us find information about influential research teams and potential collaborators, so as to identify research hotspots of interest and build partnerships.^[22,23]^

**Figure 3.**
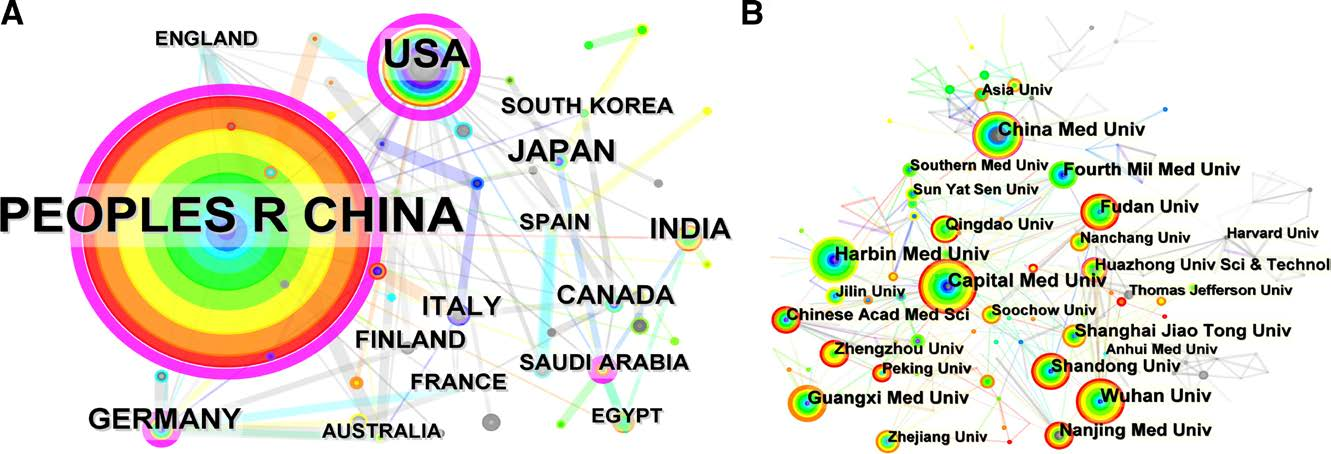
Co-occurrence maps (A) Countries; (B) Institutions.

After running VOSviewer and Citespace software, we know that 58 countries or territories have published papers, among which 30 have published more than 4 papers and 21 have published more than 7 papers, as shown in Figure 3A. The countries or territories with the largest number of publications are The People’s Republic of China (Peoples R China) (1269), followed by The United States of America (USA) (n = 329), Japan (n = 70), Germany (n = 52) and India (n = 48). Regarding the number of publications for the top 10 countries or territories, the data of citations and total link strength was outlined in Table 1. In addition, studies in this field first emerged in the USA and Japan in 1996, Canada, Italy, Finland and Spain in 1997, and even in India in 2009. The USA has the highest centrality ranking (n = 0.78), followed by Peoples R China (n = 0.41), Germany (n = 0.16), Japan (n = 0.07), Canada (0.07), and Spain (0.07).

**Table 1:** Top 10 productive countries/territories related to research.

Rank	Country	Year	Publications (%)	Centrality	Citations	Total link strength
1	Peoples R China	2000	1269 (67.5)	0.41	22,252	132
2	USA	1996	329 (17.5)	0.78	24,269	150
3	Japan	1996	70 (3.7)	0.07	3620	25
4	Germany	1998	52 (2.8)	0.16	2540	26
5	India	2009	48 (2.6)	0.01	1090	25
6	Canada	1997	44 (2.3)	0.07	2180	26
7	Italy	1999	34 (1.8)	0.06	2396	19
8	Finland	1997	25 (1.3)	0	2214	5
9	Spain	1997	19 (1.0)	0.07	743	2
10	South Korea	2003	18 (1.0)	0.03	506	8

Analysis of published institutions found that a total of 1648 institutions conducted research in the field, including 118 that published more than 6 papers and 35 that published more than 15 papers. The Capital Medical University (n = 56) and Harbin Medical University (n = 56) produced most articles, followed by China Medical University (n = 53), Wuhan university (n = 49), Guangxi Medical University (n = 43), and Fourth Military Medical University (n = 42). The China Medical University has the highest centrality (n = 0.13), while the rest of the institutions have a centrality less than 0.1. In addition, the top 10 institutions with the most publications belong to the Peoples R China, which is enough to prove that the cooperation between Chinese institutions is overwhelmingly close and active, which gives full play to regional advantages and elevates China’s academic status and influence in this field. The collaboration among institutions is shown in Figure 3B, and the top 10 institutions with regard to publications are sorted into Table 2.

**Table 2:** Top 10 institutions by publications.

Rank	Institutions	Year	Publications (%)	Centrality	Citations	Total link strength
1	Capital Medical University	2005	56 (3.0)	0.08	940	36
2	Harbin Medical University	2006	56 (3.0)	0.02	1341	4
3	China Medical University	2006	53 (2.8)	0.13	1160	74
4	Wuhan University	2008	49 (2.6)	0.05	1117	32
5	Guangxi Medical University	2006	43 (2.3)	0.02	664	11
6	The Fourth Military Medical University	2004	42 (2.2)	0.09	1406	18
7	Fudan University	2006	40 (2.1)	0.08	701	26
8	Shanghai Jiao Tong University	2013	38 (2.0)	0.05	1297	25
9	Nanjing Medical University	2004	38 (2.0)	0.02	1216	19
10	Shandong University	2014	35 (1.9)	0.03	838	15

### 3.3. Productive journals and co-cited journals

**Figure 4.**
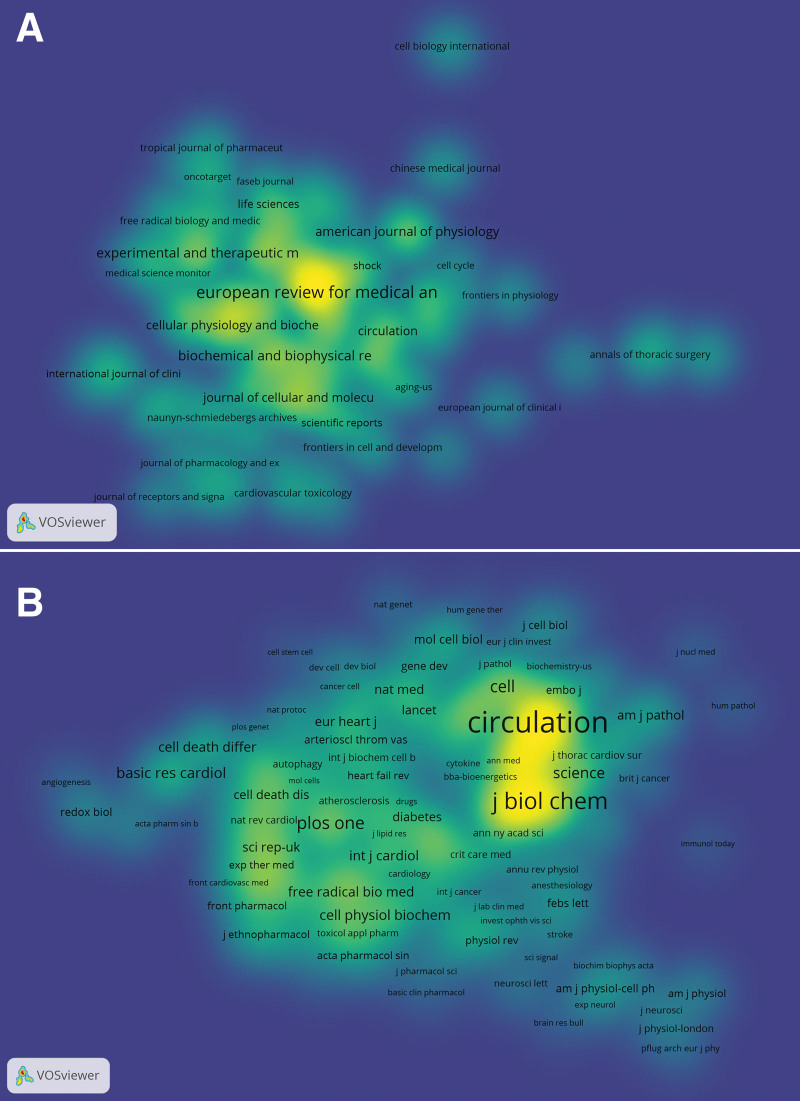
The density maps (A) Journals; (B) Co-cited journals.

We conducted journal and co-cited journal analysis to determine the most published and co-cited journals on cardiomyocyte apoptosis, we. The results of software operation showed that 1881 articles were published in various scientific journals (n = 517). Table 3 lists the top 10 journals and co-cited journals associated with myocardial apoptosis. The journal with the most publications was EUROPEAN REVIEW FOR MEDICAL AND PHARMACOLOGICAL SCIENCES (n = 73), followed by MOLECULAR MEDICINE REPORTS (n = 54), INTERNATIONAL JOURNAL OF CLINICAL AND EXPERIMENTAL MEDICINE (n = 36), EXPERIMENTAL AND THERAPEUTIC MEDICINE (n = 36), and BIOCHEMICAL AND BIOPHYSICAL RESEARCH COMMUNICATIONS (n = 35). Among all the journals included, journals with more than 4 publications were selected for density mapping (Fig. 4A) to show more clearly the high-yielding journals.

**Table 3:** The top 10 journals and co-cited journals related to myocardial apoptosis.

Rank	Journal	Publications (%)	Citations	Total link strength	Co-cited journal	Citations	Total link strength
1	European Review for Medical and Pharmacological Sciences	73 (3.9)	698	83	Circulation	3541	129,979
2	Molecular Medicine Reports	54 (2.9)	746	98	Circulation Research	2743	107,987
3	International Journal of Clinical and Experimental Medicine	36 (1.9)	162	63	Journal of Biological Chemistry	2060	84,296
4	Experimental and Therapeutic Medicine	36 (1.9)	347	50	Cardiovascular Research	1819	71,538
5	Biochemical and Biophysical Research Communications	35 (1.9)	912	76	Journal of Molecular and Cellular Cardiology	1706	67,220
6	APOPTOSIS	34 (1.8)	1253	100	American Journal of Physiology-heart and Circulatory Physiology	1626	61,988
7	American Journal of Physiology-heart and Circulatory Physiology	31 (1.7)	2544	162	Journal of Clinical Investigation	1293	51,673
8	Cardiovascular Research	27 (1.4)	2476	154	Proceedings of the National Academy of Sciences of the United States of America	1216	51,452
9	Cellular Physiology and Biochemistry	26 (1.4)	963	73	Journal of the American College of Cardiology	954	34,980
10	Journal of Cellular and Molecular Medicine	26 (1.4)	503	68	PLOS ONE	921	30,020

Total 4578 journals were cited, among them 465 were cited more than 19 times. The density map in Figure 4B shows journals with co-citations greater than 19 times. According to Table 2, the journal with the highest citation frequency is CIRCULATION (n = 3541), followed by the CIRCULATION RESEARCH (n = 2743), JOURNAL OF BIOLOGICAL CHEMISTRY (n = 2060), CARDIOVASCULAR RESEARCH (n = 1819), and JOURNAL OF MOLECULAR AND CELLULAR CARDIOLOGY (n = 1706).

**Figure 5.**
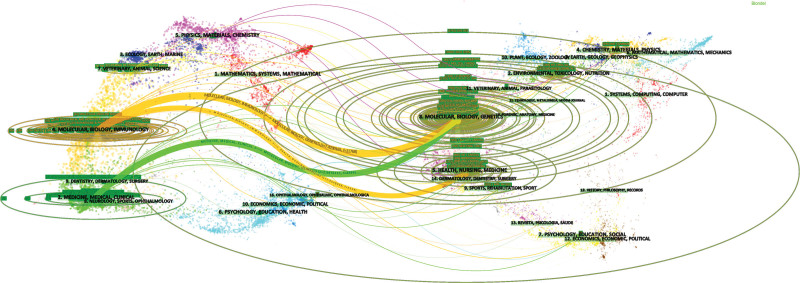
The dual-map overlay of journals related to myocardial apoptosis. Notes: Left: the citing journals, Right: the cited journals.

The dual-map overlay of journals, defined by the citing journals on the left and the cited journals on the right, is a great illustration for topic distribution of academic journals. The relationship between citing and cited journals is indicated by the colored paths. According to Figure 5, the 3 main reference paths suggest that the studies published in the journals of Health/Nursing/Medicine and Molecular/Biology/Genetics were mainly cited by the journals of Medicine/Medical/Clinical and Molecular/Biology/Immunology.

### 3.4. Authors and cited authors analysis

**Figure 6.**
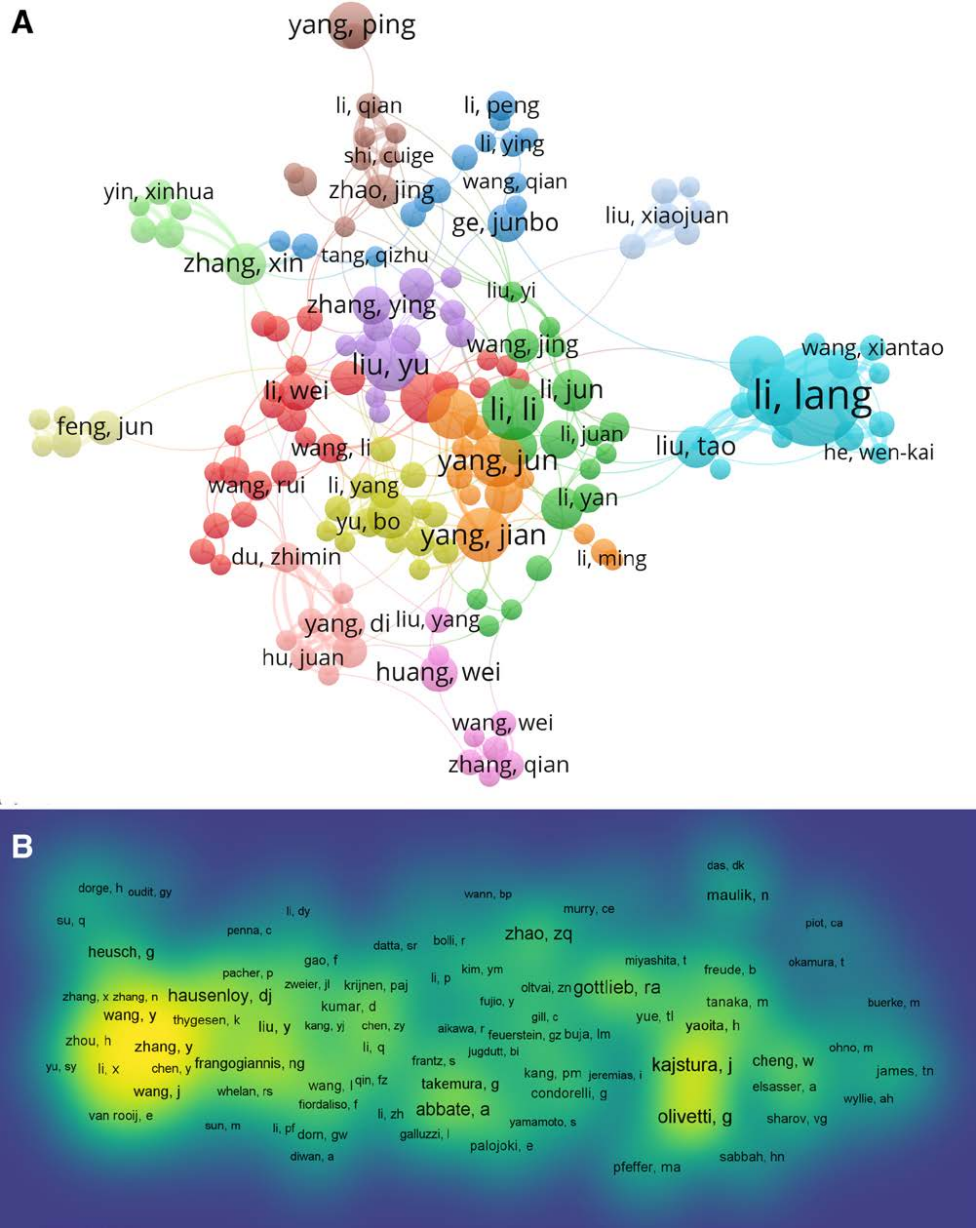
The co-occurrence maps in the myocardial apoptosis (A) Authors; (B) Co-cited authors.

Based on the operation results of VOSviewser software, 10,313 authors were found to have published relevant literatures on cardiomyocyte apoptosis. As shown in Table 4, Li Lang is the authors with the most publications (n = 24), followed by Huang Chih-Yang (n = 17), Kuo Wei-wen(n = 17), Li Li(n = 14) and Su Qiang (n = 13). Meanwhile, using the threshold of minimally 4 documents of an author, in the end, a total number of 242 authors were selected to draw the network map. As revealed in Figure 6A different colors representing different clusters, the result showed that there was a close relationship among clusters, such as Liu Yu and Li Wei, Yang Jun and Li Li, Wang Li and Li Yang. In addition, active collaborations between authors in the same cluster, such as Zhang Xin and Yin Xin-hua, Wang Wei and Zhang Qian, Li Lang and Liu Tao, were easily identified particularly.

**Table 4:** Top 10 authors by publication or cited authors by count.

Rank	Author	Publications	Citations	Total link strength	Cited author	Citations	Total link strength
1	Li Lang	24	372	70	Kajstura J	212	2147
2	Huang Chih-yang	17	290	64	Clivetti G	208	2207
3	Kuo Wei-wen	17	400	61	Saraste A	207	2027
4	Li Li	14	180	14	Gottlieb Ra	206	1845
5	Su Qiang	13	254	42	Narual J	195	1928
6	Zhou Yao	12	133	36	Hausenloy Dj	170	840
7	Yang Jian	12	490	32	Abbate A	160	1236
8	Saraste A	12	1703	27	Zhao Zq	156	1153
9	Yang Jun	12	379	22	Zhang Y	107	374
10	Liu Yu	12	339	16	Cheng W	102	1340

Co-cited authors are defined as 2 or more authors who have been simultaneously co-cited in a series of studies. A total number of 32,421 coauthors were detected. As summarized in Table 4, all of the top 10 authors were co-cited by more than 100 times, the most frequently one was Kajstura J (n = 212), followed by Clivetti G (n = 208), Saraste A (n = 207), Gottlieb Ra (n = 206), and Narual J (n = 195). In addition, the authors (n = 292) with more than 20 co-citations were included in the density map, which is a well depiction of high-frequency co-cited authors according to the gradation of yellow (Fig. 6B).

### 3.5. Keyword co-occurrence, clusters, and evolution

**Figure 7.**
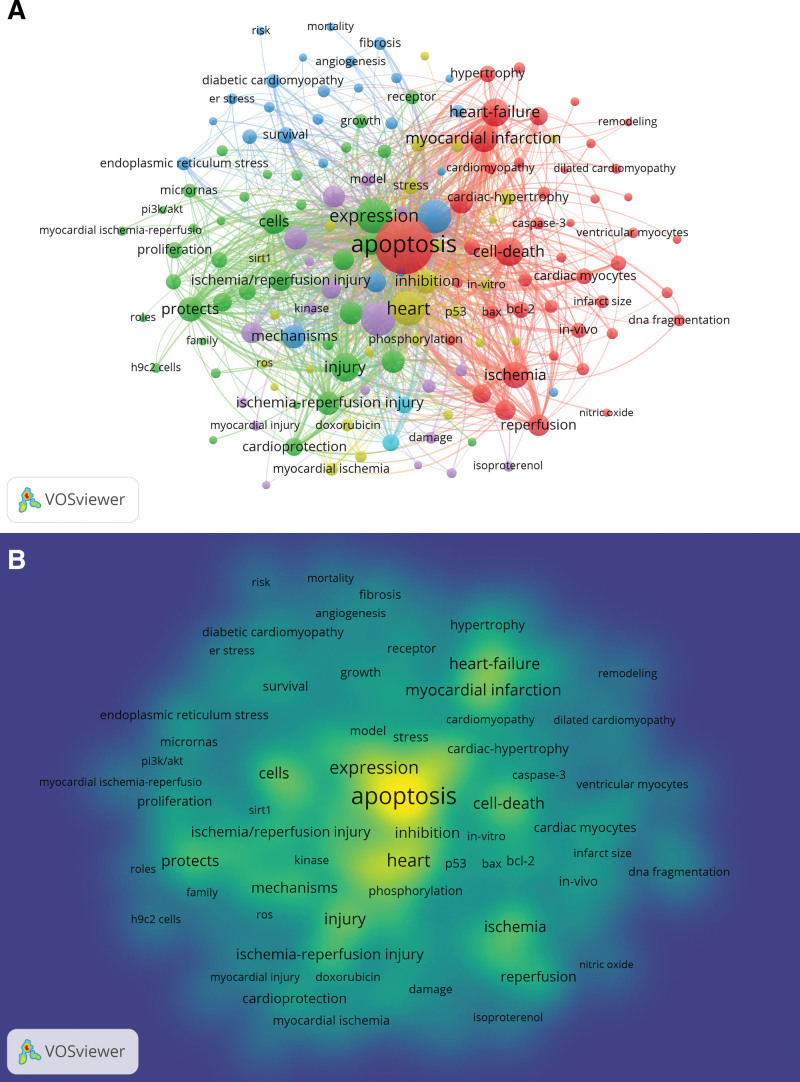
Maps of keywords in the myocardial apoptosis (A) Co-occurrence network and clusters; (B)The density map.

Keywords are the high generalization of a thesis theme and the core of an article.^[24,25]^ Therefore, by analyzing the keywords of a paper, we can understand the themes and key points of the paper, and find the research status and hot trends.^[26]^ VOSviewer offers keyword co-occurrence and network clustering analysis. Using this software, about 5677 keywords were extracted. Considering the small influence on a few occurrence of the keyword, the chosen threshold was set with “minimum number of occurrences of a keyword ≥ 20.” Finally, only 115 keywords fit the threshold selection, as shown in Figure 7A, which also presented network clustering analysis on keywords. The 6 clusters in this map represent 6 directions and scopes of research, in which cluster 1 (red color) was largest, which was in turn followed by cluster 2 (green), cluster 3 (dark blue), cluster 4 (yellow), cluster 5 (purple), and cluster 6 (wathet blue). In cluster 1, it is intriguing to note the presence of a total of 44 different items in Cluster 1, including apoptosis, cell-death, heart-failure, hypertrophy, remodeling, bax, bcl-2, caspase-3, ischemia, reperfusion, ventricular myocytes, and others. Similarly, Cluster 2 comprises 38 items, such as proliferation, injury, cardioprotection, receptor, expression, and others. Besides, Cluster 3 encompasses 26 items, including fibrosis, angiogenesis, risk, survival, mortality, diabetic cardiomyopathy, and others. Furthermore, Cluster 4 includes phosphorylation, myocardial ischemia, doxorubicin, ros, inhibition, and others. Notably, And, Cluster 5 exhibits distinct items, such as kinase, model, myocardial injury, isoproterenol, and other 19 items. Finally, Cluster 6 consists of 2 items, i.e. ischemia and reperfusion. The 20 keywords with the highest frequency are derived (Table 5), which represent the hotspots of the myocardial apoptosis. Meanwhile, there are 10 keywords that appear more than 200 times. Ranking based on frequency, the keyword “apoptosis” ranked first (n = 1032), followed by “expression” (n = 372), “heart” (n = 364), “oxidative stress” (n = 354), “activation” (n = 347), “cell-death” (n = 252), “injury” (n = 248), “heart-failure” (n = 242), “myocardial infarction” (n = 223), and “ischemia” (n = 206). Lastly, in the density map, these high-frequency keywords were intuitively depicted (Fig. 7B).

**Table 5:** The top 20 keywords associated with the myocardial apoptosis.

Rank	Keywords	Count	Rank	Keywords	Count
1	apoptosis	1032	11	cells	193
2	expression	372	12	protects	186
3	heart	364	13	inhibition	174
4	oxidative stress	354	14	mechanisms	167
5	activation	347	15	cardiomyocyte apoptosis	166
6	cell-death	252	16	ischemia-reperfusion injury	165
7	injury	248	17	infarction	161
8	heart-failure	242	18	inflammation	161
9	myocardial infarction	223	19	cardiomyocytes	156
10	ischemia	206	20	dysfunction	150

**Figure 8.**
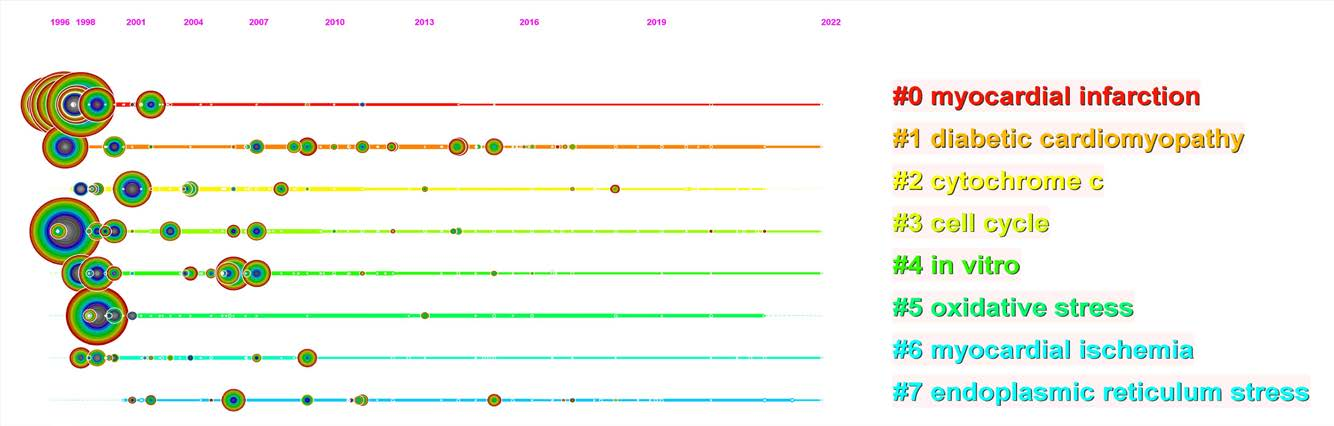
Timeline view of keywords.

CiteSpace is used to build a keyword timeline viewer, that can cluster keywords and at the same time consider the time factor, which is a good illustration of high-frequency keywords evolved in each cluster. In addition, the readers can assist in locating the period of a particular subject and the evolution trajectory of field of study. As shown in Figure 8, the log-likelihood ratio method was used to divide the keywords into 8 categories, which can visually see the research focus and evolution trajectory of cardiomyocyte apoptosis in each stage. In addition, the exported clustering data was shown in Table 6.

**Table 6:** Keyword clustering data sheet.

ClusterID	Size	Silhouette	Mean (year)	Label (LLR)
#0	98	0.723	2000	Myocardial infarction; acute myocardial infarction; programmed cell death
#1	72	0.597	2012	Diabetic cardiomyopathy; cell apoptosis; angiogenesis
#2	71	0.661	2009	Cytochrome c; endothelial cell; myocardial injury
#3	64	0.664	2011	Cell cycle; receptor; fibrosis
#4	60	0.664	2009	In vitro; hypoxia/reoxygenation injury; ischemia/reperfusion injury
#5	55	0.751	2005	Oxidative stress; coronary microembolization; lipid peroxidation
#6	50	0.69	2009	Myocardial ischemia; myocardial remodeling; cleavage
#7	49	0.633	2015	Endoplasmic reticulum stress; perk; toxicity

**Figure 9.**
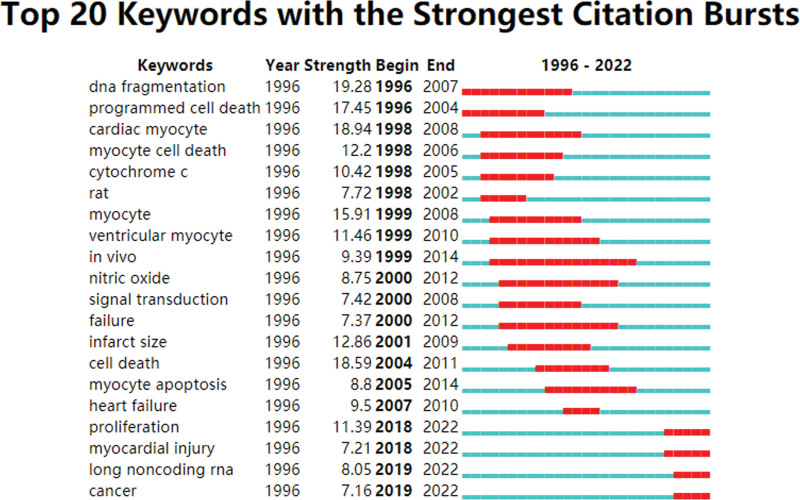
Top 20 keywords with the most frequent citation bursts.

Burst detection is an algorithm that determines the change of variables within a pre-defined time window. The burst detection of keywords can reflect the research trend in a certain stage and predict the future research direction according to the duration of hotspots.^[25,27]^ The index for measuring the intensity of the citation outbreak is named the intensity value: the larger the value, the more intense the outbreak. We set γ = 1.0 with a minimum duration of 3 years, 61 keywords were detected and the first 20 were displayed as shown in Figure 9. The intensity of the keywords ranged from 7.16 to 19.28, and the duration of the burst ranged from 3 to 15 years. Among them, DNA fragmentation^[19,28]^ has the highest intensity, while in vivo (15 years) has the longest duration. The keywords of proliferation, myocardial injury, long noncoding rna and cancer continue to this day, indicating that they are still the focus and direction of future research.

## 4. Discussion

### 4.1. General information

The trend of annual publication indicates that the research field under discussion has garnered significant attention from researchers since 1996 and continues to be a prominent and active area of investigation. With more in-depth research published in this field, the number of literatures are increasing. However, there are still many key scientific problems to be solved. Therefore, it is still a promising research hotspot, which is worth investing more manpower, material and financial resources. Currently, there is a need for enhanced collaboration and cooperation among countries/territories and institutions, as close ties have yet to be fully established. Therefore, it is necessary to eliminate academic barriers, establish more in-depth and extensive cooperation, exchanges between countries and institutions further promote the overall development of the field and provide better clinical care for more patients. Examination of published journals further revewals a focus on both basic research and translational medicine. Analyzing the authors involved allows for the identification of potential collaborators and influential research groups.

### 4.2. The hotspots and frontiers

Keywords co-occurrence analysis of bibliometrics could imply the hotspots and directions of a particular research field. As can be seen from Table 5, the top 20 high-frequency keywords involved in cardiomyocyte apoptosis included apoptosis, oxidative stress, activation, heart-failure, myocardial infarction, mechanisms, inflammation and dysfunction. From these representative terms, an overview of the research field can be summarized. It is manifested in the following 3 aspects:

(1) The commonly used research objects are cells, rats and mice, while human research objects are rarely used.

Through the analysis of keywords, we found that the frequency of cells, rats and mice was higher, indicating that the commonly used research objects in this field are these 3 kinds. A subsequent reading of the titles and abstracts of the included literature confirmed this inference. Deng et al^[28]^ used H9c2 cells and discovered that the noncoding repressor of nuclear factor of activated T cells up-regulated the expression of si-hypoxia-inducible factor-1 alpha (HIF-1 alpha), thereby alleviating cardiomyocyte apoptosis induced by hypoxia/ reoxygenation (H/R). Wei et al^[29]^ employed Doxorubicin (DOX) to establish a rat heart injury model and found that Wogonin protected the rat heart from DOX-induced damage by inhibiting the release of mitochondrial cytochrome c and reducing the apoptosis of cardiomyocytes caused by caspase activation. Xu et al,^[30]^ utilizing male C57/B6J mice, found that Shenfu injection up-regulated the expression of B-cell lymphoma-2 (Bcl-2) protein, thereby inhibiting myocardial apoptosis and mitigating mitochondrial damage induced by septicemia through downregulation of BH3 interacting domain death agonist and caspase-9 proteins.

In summary, the subjects in this field are mainly cells, rats and mice, and the modeling methods include surgery (coarctation of aorta, ligation of left anterior descending branch, coarctation of abdominal aorta) and drugs (doxorubicin, isoproterenol, angiotensin II). It is essential for researchers to actively establish a standardized diagnosis and treatment system, strengthen evidence-based evidence collection, and carry out high-quality multi-center, large sample randomized double-blind clinical trials.

(2) Diseases associated with cardiomyocyte apoptosis include heart failure, myocardial infarction, and ischemia-reperfusion injury.

Cardiomyocyte apoptosis is an important pathological link of heart failure, myocardial infarction, ischemia-reperfusion injury and other cardiovascular diseases. Numerous studies have consistently demonstrated that increased apoptosis contributes to the development of myocardial infarction and heart failure, whereas interventions aimed at limiting apoptosis can provide protection for the heart.^[5]^ For instance, Li et al^[31]^ found that under the stimulation of H/R or DOX, miR-181c can down-regulate the protein expression of Fas, IL-6 and Tnf-α, and up-regulate the phosphorylation of Bcl-2 and Akt. These findings suggest that miR-181c may inhibit cardiomyocyte apoptosis and alleviate heart failure through PI3K/Akt pathway.

In conclusion, myocardial apoptosis dysregulation leads to a variety of diseases, and modulation of myocardial apoptosis may serve as a therapeutic target for its related diseases. It is important to study the pathway and mechanism of cardiomyocyte apoptosis and develop related drugs for the treatment of such diseases.

(3) The relevant pathological mechanisms include oxidative stress, inflammatory response, cardiac hypertrophy and autophagy.

Oxidative stress, inflammation, cardiac hypertrophy and autophagy are closely related to apoptosis. Previous publications demonstrated highlighted oxidative stress as one of the key determinants in the balance between cardiomyocyte apoptosis and survival.^[32]^ Oxidative stress leads to mitochondrial DNA oxidation and disruption of protein functions, resulting in significant mitochondrial damage.^[33]^ Consequently, the Caspases are activated and apoptosis is triggered.^[34]^ Furthermore, a number of circulating miRNAs might be used as potential biomarkers for reactive oxygen species-related cardiac diseases.^[35]^ It is reported that miR-370 diminished the oxidative stress and prevented apoptosis of cardiomyocytes in the presence of hydrogen peroxide.^[36]^ In addition, inhibition of miR-205 suppresses cardiac I/R injury by regulating oxidative stress and apoptosis.^[37]^ Inflammation also plays a role in inducing apoptosis. Notably, apoptosis is now considered an important mechanism in the resolution of inflammation. Studies have shown that upregulation of miR-425-3p reduced cardiomyocyte apoptosis and alleviate myocardial inflammation in mice with viral myocarditis, thus improving their survival rates.^[38]^ In addition, the relationship between autophagy and apoptosis is complex. Upon starvation or other stress conditions, autophagy is induced, and when stress persists, apoptotic are activated. An inhibition of caspase-dependent apoptosis enhanced autophagy, and an inhibition of autophagy also enhanced caspase activation.^[39]^

Overall, these pathological mechanisms influence each other and interact with each other instead of existing in isolation. In-depth study of the relationship between various pathological mechanisms is helpful to guide treatment. strategies Based on our analysis and hypothesis, experiments should be designed reasonably in the future to comprehensively study the cross-talk between different pathological mechanisms, allowing for deeper insights into pathogenesis and development of more effective therapeutic interventions.

### 4.3. Strength and limitation

In cardiovascular science, myocardial apoptosis has a tremendous research value and clinical potential. Using CiteSpace analysis can better identify research priorities and trends, compared with traditional reviews.

But it also has certain limitations. CiteSpace could not be used to entirely replace system retrieval. First of all, the collected literature data is inconsistent, which will damage the credibility of the atlas rendering. Second, the difference in data updating will cause discrepancy between the retrieval results and the actual number of articles included. Second, when analyzing authors using dedicated software, it is observed that an author with a single first author publications typically attains a lower ranking compared to authors with 2 publications as a middle authorship. However, it is well acknowledged that the publication of the first author carries more prestige and significance than that of the intermediate author. Nevertheless, the literature analysis based on visualization is advantageous in providing better understanding on the most authoritative literature in the research area, and it could also establish a foundation for subsequent research.

## 5. Conclusion

In this study, 1881 literatures in the field of cardiomyocyte apoptosis were visualized and analyzed bibliometrically by adopting CiteSpace and VOSviewer software. Our results show that the number of publications increases annually, indicating that this field has attracted more and more attention and has a broad research prospect. China and the United States are the major players engaged in this research area, but exchanges between different countries, regions and institutions need to be further reinforced to jointly promote development and progress in this research area. The commonly used research objects are cells, rats and mice. Research hotspots mainly focus on clinical diseases including heart failure and myocardial infarction, and microscopic mechanisms such as oxidative stress, autophagy, inflammatory response and cellular pathways.

## Acknowledgments

The authors would like to thank Hunan University of Chinese Medicine for its support of this work, and the editors and reviewers for their valuable comments and permission to improve the manuscript.

The authors thank 51runse (www.51runse.cn) for the English language editing during the preparation of this manuscript.

## Author contributions

**Conceptualization:** Kun Lian, Xin Li, Meng Yang, Jiahao Ye, Zhixi Hu.

**Data curation:** Kun Lian, Xin Li, Xiaoyi Wang, Fei Wang, Jiahao Ye.

**Formal analysis:** Kun Lian, Meng Yang, Zhixi Hu.

**Funding acquisition:** Lin Li, Zhixi Hu.

**Investigation:** Kun Lian, Xin Li.

**Methodology:** Kun Lian, Xiaoyi Wang, Meng Yang.

**Project administration:** Kun Lian, Fei Wang, Lin Li, Zhixi Hu.

**Resources:** Xiaoyi Wang, Meng Yang, Lin Li.

**Software:** Kun Lian, Xin Li, Xiaoyi Wang, Meng Yang.

**Supervision:** Kun Lian, Jiahao Ye, Lin Li, Zhixi Hu.

**Validation:** Kun Lian, Lin Li, Zhixi Hu.

**Visualization:** Kun Lian, Fei Wang, Jiahao Ye.

**Writing – original draft:** Kun Lian.

**Writing – review & editing:** Kun Lian.
